# Controlling the bacterial load of *Salmonella* Typhi in an experimental mouse model by a lytic *Salmonella* phage STWB21: a phage therapy approach

**DOI:** 10.1186/s12866-023-03040-3

**Published:** 2023-11-03

**Authors:** Payel Mondal, Prolay Halder, Bani Mallick, Subhadip Bhaumik, Hemanta Koley, Shanta Dutta, Moumita Dutta

**Affiliations:** 1 Division of Electron Microscopy, ICMR-National Institute of Cholera & Enteric Diseases, P-33, C.I.T. Road, Scheme XM, Beliaghata, 700010 Kolkata, West Bengal India; 2 Division of Bacteriology, ICMR-National Institute of Cholera & Enteric Diseases, P-33, C.I.T. Road, Scheme XM, Beliaghata, 700010 Kolkata, West Bengal India; 3 University Science Instrumentation Centre, The University of Burdwan, Golapbag, Burdwan, 713104 West Bengal India

**Keywords:** *Salmonella* Typhi, Lytic phage, In vivo, Animal model, Phage therapy

## Abstract

**Background:**

*Salmonella enterica* serotype Typhi is one of the major pathogens causing typhoid fever and a public health burden worldwide. Recently, the increasing number of multidrug-resistant strains of *Salmonella spp*. has made this utmost necessary to consider bacteriophages as a potential alternative to antibiotics for *S.* Typhi infection treatment. *Salmonella* phage STWB21, isolated from environmental water, has earlier been reported to be effective as a safe biocontrol agent by our group. In this study, we evaluated the efficacy of phage STWB21 in reducing the burden of salmonellosis in a mammalian host by inhibiting *Salmonella* Typhi invasion into the liver and spleen tissue.

**Results:**

Phage treatment significantly improved the survival percentage of infected mice. This study also demonstrated that oral administration of phage treatment could be beneficial in both preventive and therapeutic treatment of salmonellosis caused by *S.* Typhi. Altogether the result showed that the phage treatment could control tissue inflammation in mice before and after *Salmonella* infection.

**Conclusions:**

To the best of our knowledge, this is the first report of phage therapy in a mouse model against a clinically isolated *Salmonella* Typhi strain that includes direct visualization of histopathology and ultrathin section microscopy images from the liver and spleen sections.

## Introduction

*Salmonella enterica*, a Gram-negative bacterium that belongs to the Enterobacteriaceae family, is generally divided into typhoidal *Salmonella* (TS) and non-typhoidal *Salmonella* (NTS) serotypes based on clinical symptoms [1]. A potentially fatal multisystemic infection, typhoid fever or enteric fever, is primarily produced by the human-adapted pathogen *Salmonella enterica* serovar Typhi and significantly contributes to global morbidity and mortality in low-and middle-income countries [2]. It is a common foodborne pathogen that is primarily present in poultry, eggs, and dairy products [3]. It may be transferred by human feces, contaminated food, water, and person-to-person contact. The fecal-oral route is the most prevalent way of transmission [4]. The risk of morbidity and death associated with typhoid fever can be decreased by treating the condition with appropriate antibiotics.

During acute infection of *S.* Typhi shedding, the dosage and duration are important determinants in antibiotic therapy [5]. According to the WHO priority list of pathogens, *Salmonella* belongs to the highest priority pathogens group that needed attention to search for new antibiotics. Fluoroquinolone, ampicillin, trimethoprim-sulfamethoxazole, and chloramphenicol made up the conventional typhoid fever treatment regimens [6, 7]. Nevertheless, most of the bacterial strains are now found to be resistant due to the development of multidrug-resistance mechanisms. The percentage of typhoid fever isolates that were nalidixic acid-resistant was reported to be over 60% in Kolkata [8]. As a result of this, third-generation cephalosporins are now being used more often. Moreover, first- and second-line antibiotics and both fluoroquinolones and third-generation cephalosporins-resistant *Salmonella* strains have been reported in Nepal and Eastern India [9–11]. The World Health Organization (WHO) has given an estimate that by 2050 drug-resistant infections will kill millions of people every year [12]. The increase in treatment cost due to the unavailability of a suitable antibiotic will create a huge economic burden and may push millions of people into acute poverty [13]. This is definitely minacious to mankind and requires to be handled with an unconventional yet affordable treatment strategy such as phage therapy [9, 14].

Bacteriophages are bacterial viruses, abundant in nature, highly specific, and effective in killing their targeted host bacteria [15]. The mechanism of killing bacteria and developing bacterial resistance in the case of phage and antibiotics is fundamentally different [16–18]. The advantage of phage usage in the prevention of bacterial infections is due to their strict host specificity, high bactericidal efficiency, safety, low cost of production, and ease of extraction and preservation [19, 20]. However, the therapeutic efficacy of phage treatment has been explicitly demonstrated by only a handful of successful clinical trials that adhered to the current norms of evidence-based medicine [21]. Therefore, to conduct a trial with a greater possibility of success, it is crucial to analyse carefully the data obtained from experimental trials in animals.

In vivo investigation using laboratory animals is an essential component of research on determining the efficacy and safety of novel isolated bacteriophages [22]. An accurate assessment of the mechanism of phage therapy in living organisms can be preferred using animal models such as mouse, rat, and rabbit models because of their genetic closeness to humans. These models also provide information on the immune response (including any potential interactions with immune system cells like phagocytes), the gut microbiota, and infected tissue, as well as the extent of safety, tolerability, and observation of any possible adverse effects of the preparation being used [23, 24]. Our aim was to evaluate the efficacy of phage STWB21 in an animal model that would be advantageous in the future for the treatment of illnesses brought on by the *Salmonella* Typhi bacterium. In our previous work, we have shown *Salmonella* phage STWB21 fulfils the initial criteria to be a potential antibacterial agent [25]. In this study, we report that the phage STWB21 can control *Salmonella* invasion both prophylactically and therapeutically in mice. Additionally, we compared the preventive and therapeutic benefits of STWB21 treatment on mice against lethal *S.* Typhi infection without producing any negative effects, emphasizing the clinical importance of this phage to *S.* Typhi*-*induced illnesses.

## Result

### Phage propagation

**Fig. 1:**
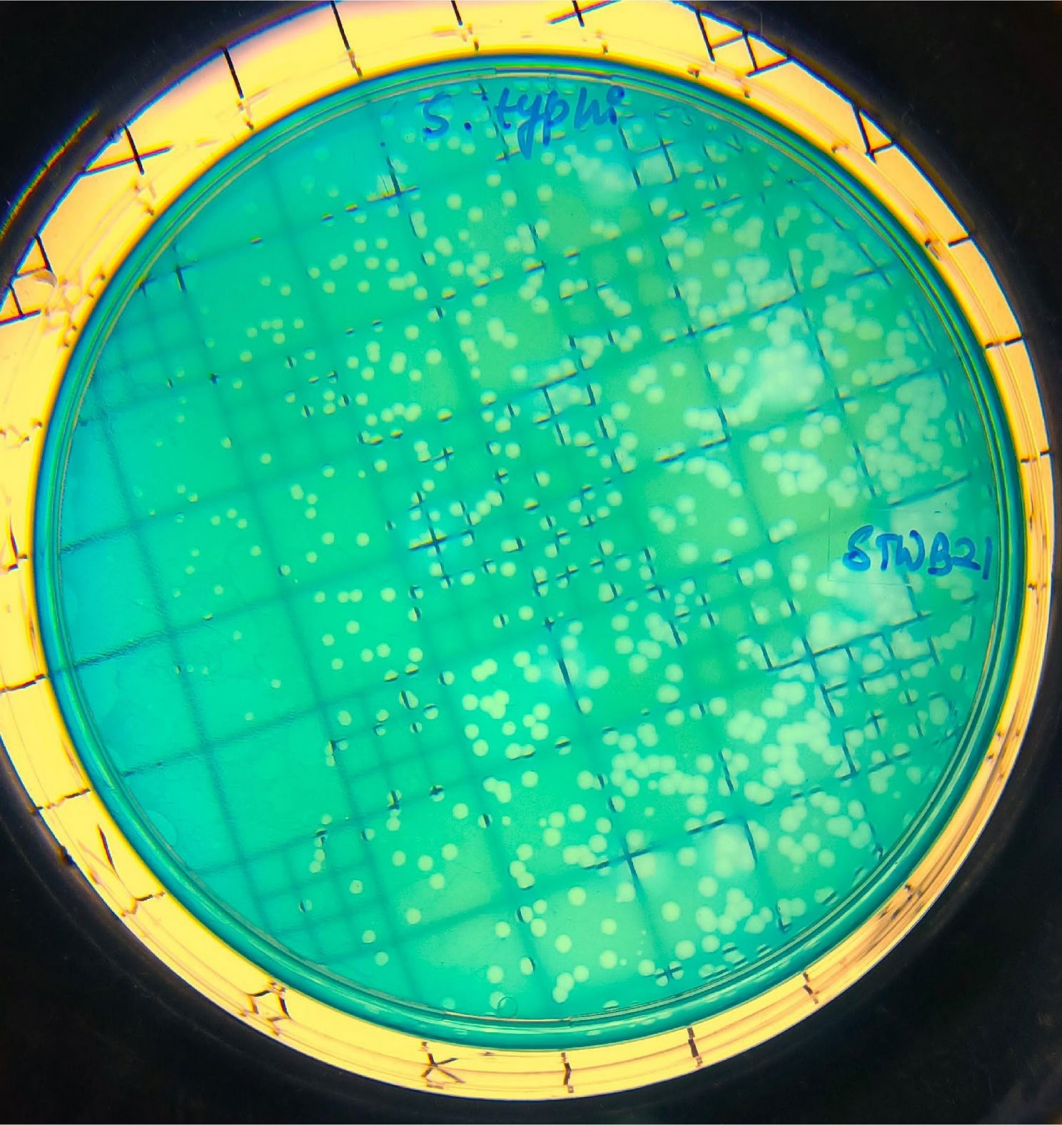
Plaques of STWB21 against *S.* Typhi *on HEA plate*

After overnight incubation, the plaques of phage on the HEA plate with *S.* Typhi bacterial lawn were measured around 1 mm in diameter. It indicates the presence of *S*. Typhi-specific phage STWB21 (Fig. 1).

### Determination of LD_50_

**Fig. 2:**
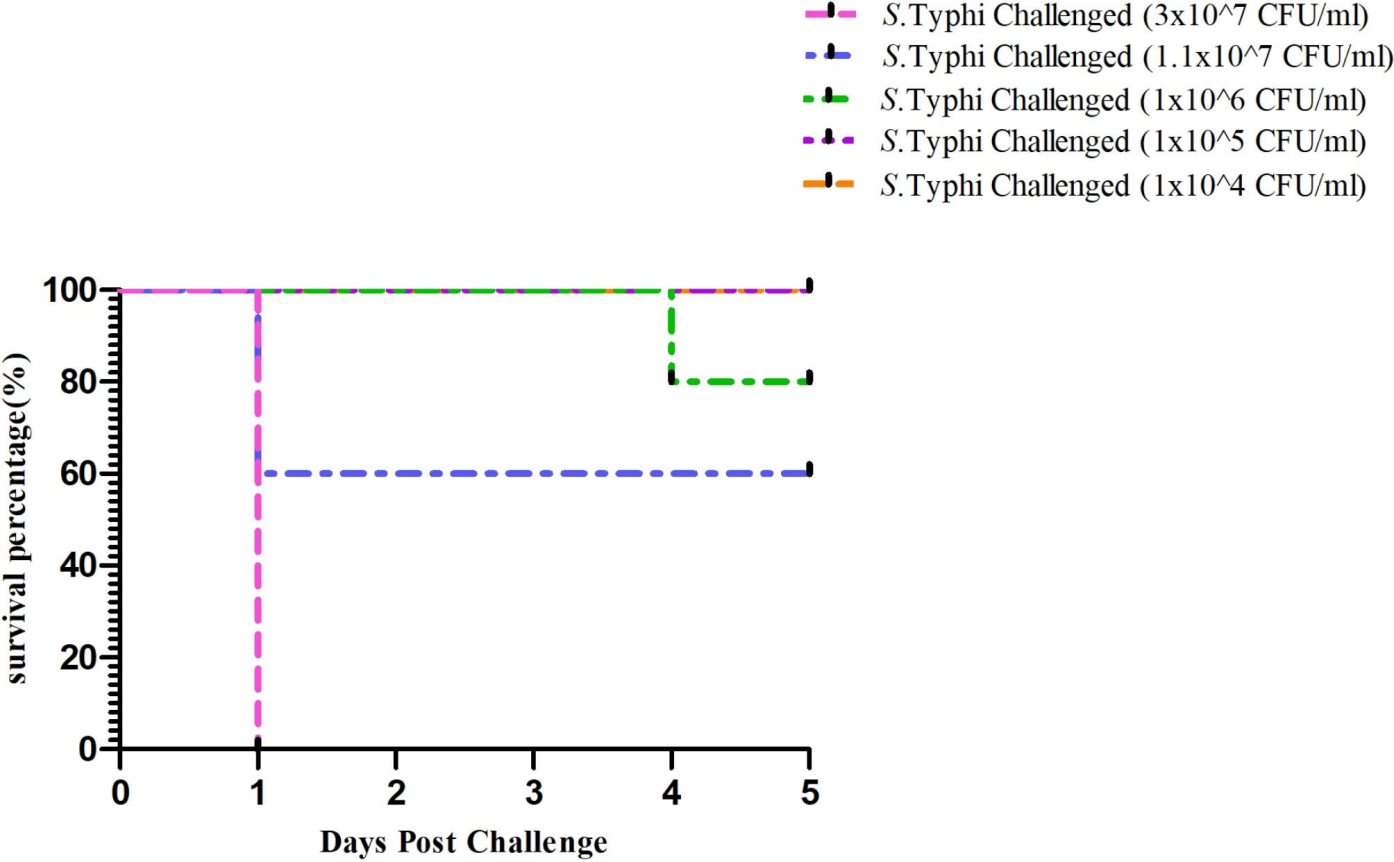
Determination of the mean lethal dose (LD_50_) of *S.* Typhi host strain in BALB/c mice n = 6; each group was intraperitoneally (i.p) infected with serially diluted bacterial suspensions of *S.* Typhi. The percentage of survival was determined for 7 days

All infected mice in the LD_50_ determination study developed illness starting at the post-challenge. Only a few mice in groups 1, 2, and 3 were still sick at the end of the experiment, and death was nil or minimal. However, significant deaths occurred in groups 4 and 5. From the post-challenge day onwards, these mice began to perish, and by the conclusion of the trial, every mouse that had survived was gravely sick. Figure 2 displays the mortality of the mice on the fifth day following infection. The mean fatal dosage was determined to be 3 × 10^7^ CFU/mice based on the observation.

### Oral treatment with phage STWB21 significantly reduced *Salmonella* proliferation in mice

**Fig. 3:**
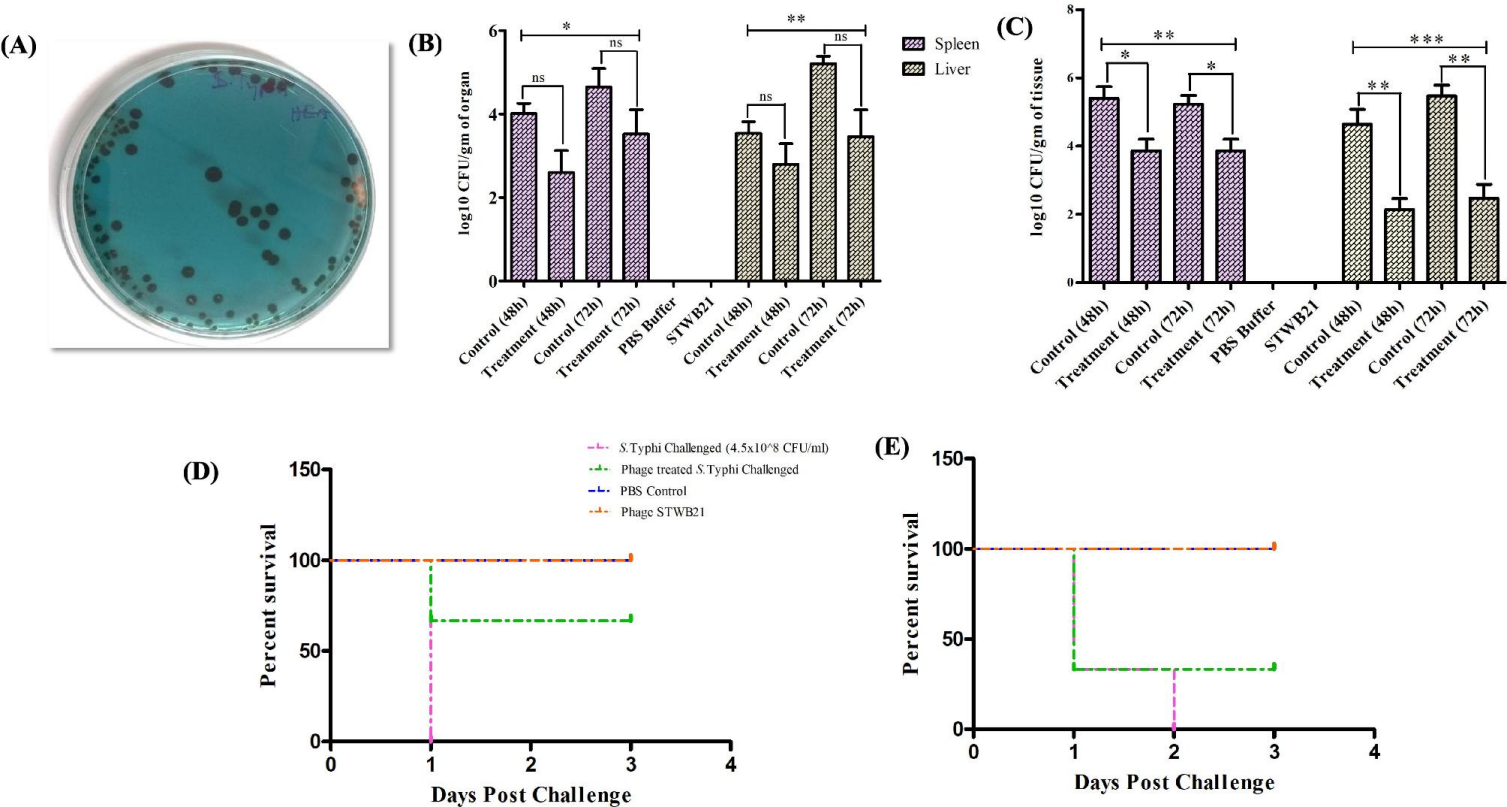
**(A)** *Salmonella* bacterial colonization on HEA plate. (**B)** Colonization of prophylactic treatment. (**C)** Colonization of therapeutic treatment. (**D)** Impact of single dose phage treatment on *S.* Typhi before infection (prophylactic treatment) and survival rates of BALB/c mice. (**E)** Impact of single dose phage treatment on *S.* Typhi after infection (therapeutic treatment) and survival rates of BALB/c mice. A statistically significant difference (*P < 0.05) was observed using 6 mice per group on phage treatment. Error bars represent the standard error of the mean (SEM) of three independent replicates and data were analyzed using GraphPad Prism 5.0

The significant effectiveness of phage STWB21 against the *S.* Typhi infection was examined in a mouse model. The effect of phage indicated decreased colonization (Fig. 3A) in both the prevention group (or prophylactic group) and the treatment group (or therapeutic group) as shown in Figs. 3B and  3C. Additionally, after observing 4 days, the survival rate of the prevention group is almost 66% (Fig. 3D) in comparison with the treated group which is almost 33% (Fig. 3E). However, the viability of the phage control group was similar to the PBS control group, indicating the safety of phage STWB21.

### Comparison of preventive versus therapeutic phage treatment using light microscopy and transmission electron microscopy

**Fig. 4:**
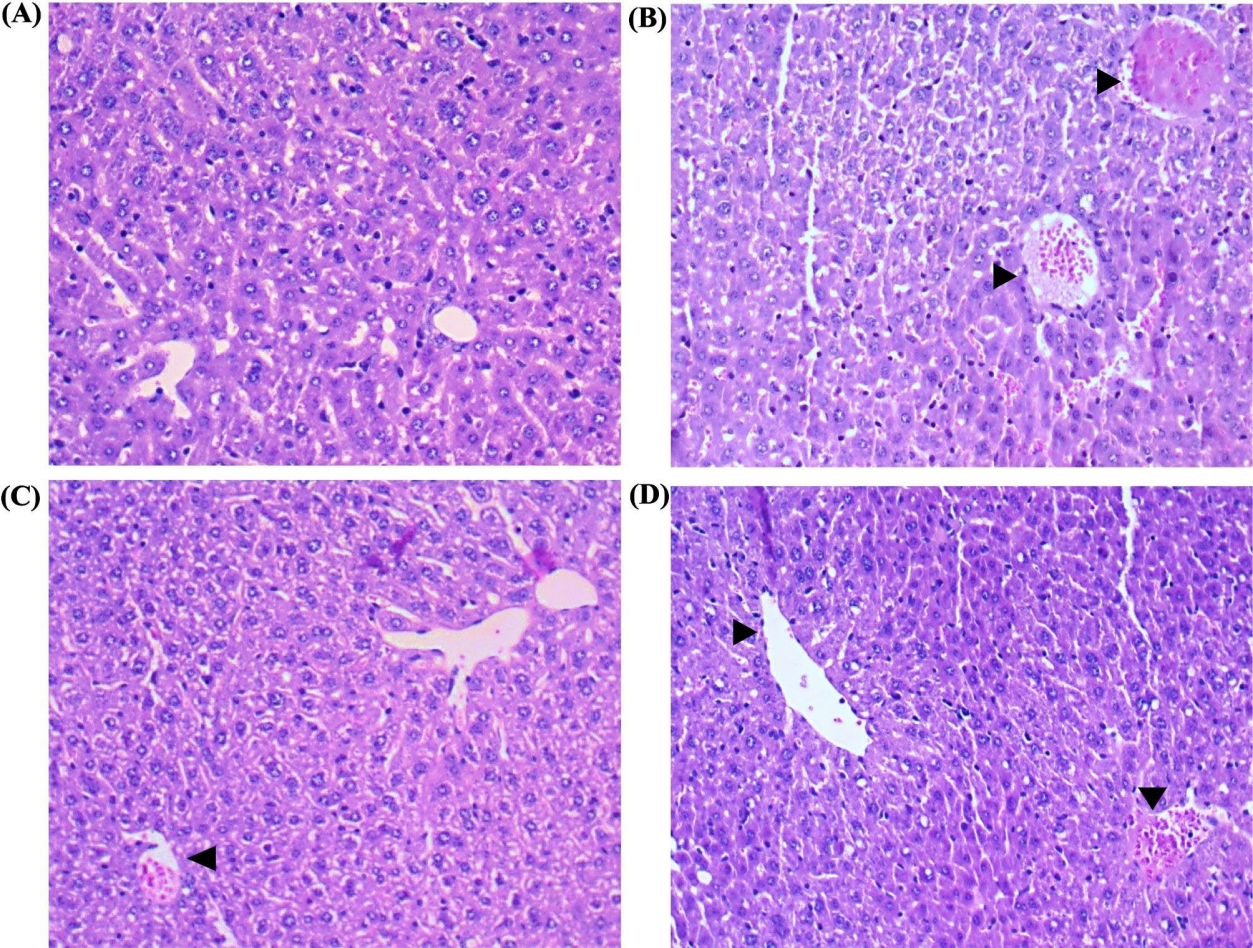
H&E staining sections of the liver from normal, infected, and treated mice. Sections were stained with hematoxylin and eosin. Magnification, X10. **(A)** Normal: without any infection or treatment. Central venule, hepatic sinusoids, plates of hepatic cells, and portal areas were seen. **(B)** Infected: mice liver at 48 h post-challenge. The central venule was inflamed. **(C)** Liver histopathological sections of Prophylactic treatment grouped mice. **(D)** Liver histopathological sections of Therapeutic treatment grouped mice

**Fig. 5:**
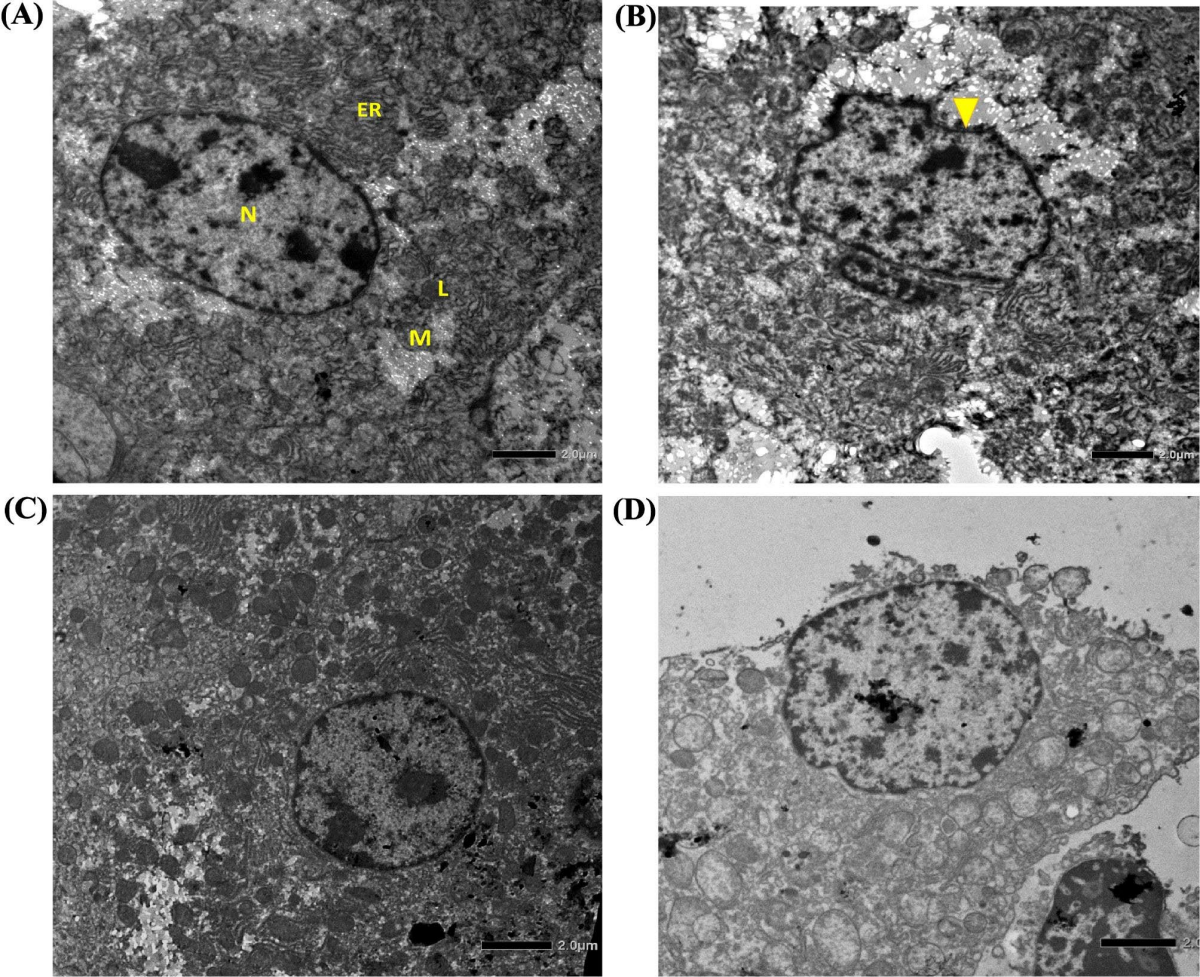
Transmission electron micrograph of mice liver sections. Magnification, X 1500. **(A)** Control: mouse liver section showing normal hepatic architecture (M: Mitochondria, N: Nucleus, L: Lysosome, ER: Endoplasmic reticulum). **(B)** Infected: mice liver at 48 h post-challenge. The central venule was inflamed. **(C)** Prophylactic treatment grouped mice. **(D)** Therapeutic treatment grouped mice

A comparative study was carried out to evaluate the effectiveness of phage STWB21 treatment prophylactically and therapeutically. Figures 4 and 5, showed the light microscopy images of histopathological sections and electron microscopic images of the stained ultrathin sections of mice liver tissue respectively. In Fig. 4A, the liver section of normal mice showed normal histology with central vein and hepatocytes arranged in the hepatic cords, and in Fig. 5A, a round nucleus (NU), normal mitochondria (MT), and endoplasmic reticulum (ER) were observed. However, in infected mice, pyknotic nuclei (PN) with the appeared nucleolus, mitochondria (M), and fragmented rough endoplasmic reticulum (RER), liver necrosis, and abscess were observed with hepatomegaly in Figs. 4B and 5B. Transmission electron micrographs of both the preventive group and therapeutic group (Fig. 5C and 5D) showed an elevation in lysosomes and mitochondria level and an increase in their density too. Interestingly, the preventive group showed minimal damage rather than the therapeutic group.

**Fig. 6:**
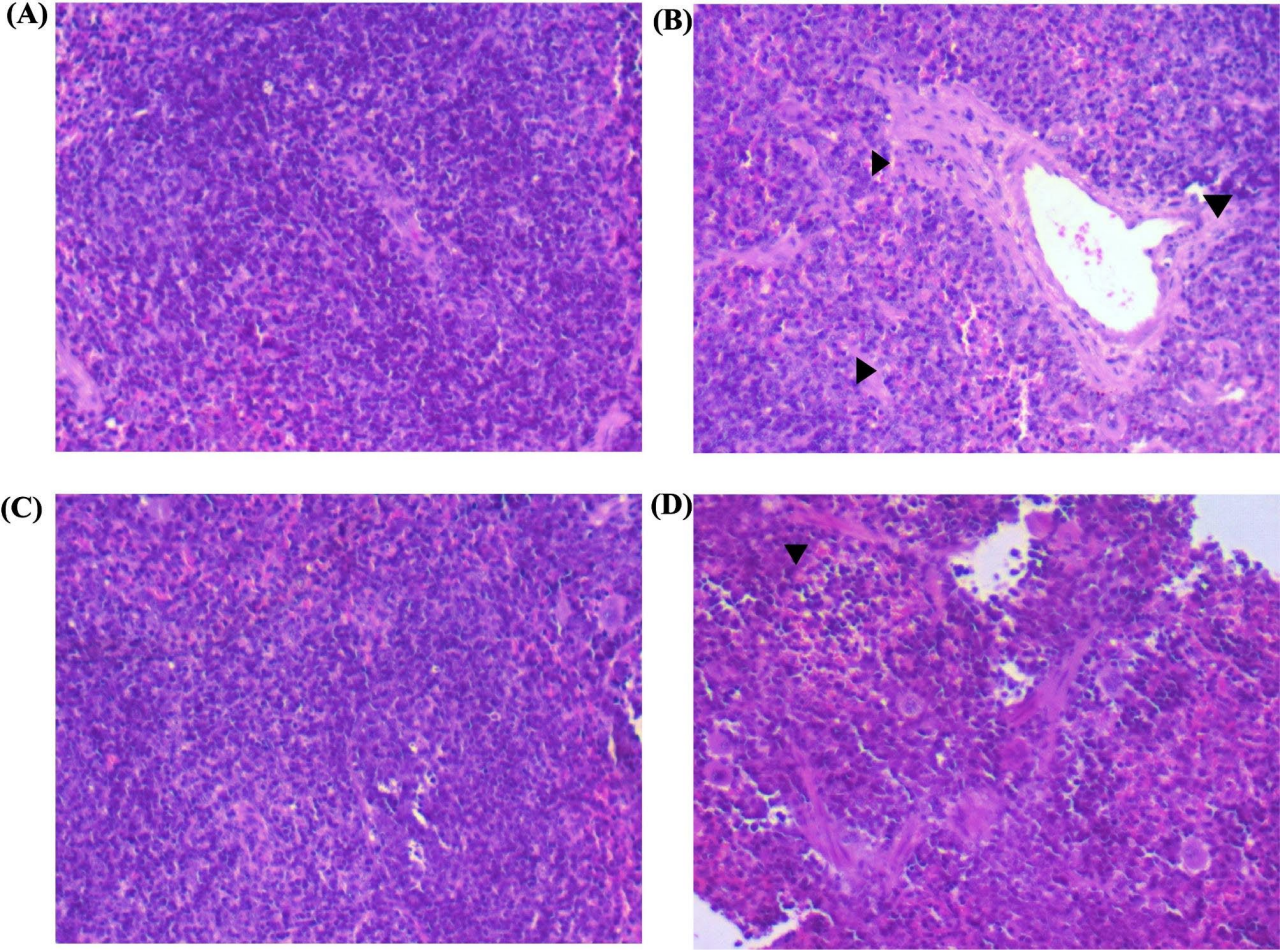
Images from the light microscope of mice spleen histopathological sections stained with hematoxylin and eosin. Magnification, X10. **(A)** Control: without any treatment. Red pulp, white pulp, plates of hepatic cells, and portal areas were visible. **(B)** Infected: mice spleen at 48 h post-challenge. The central venule was inflamed. **(C)** Spleen histopathological sections of preventive grouped mice. **(D)** Spleen histopathological sections of therapeutic treatment grouped mice

**Fig. 7:**
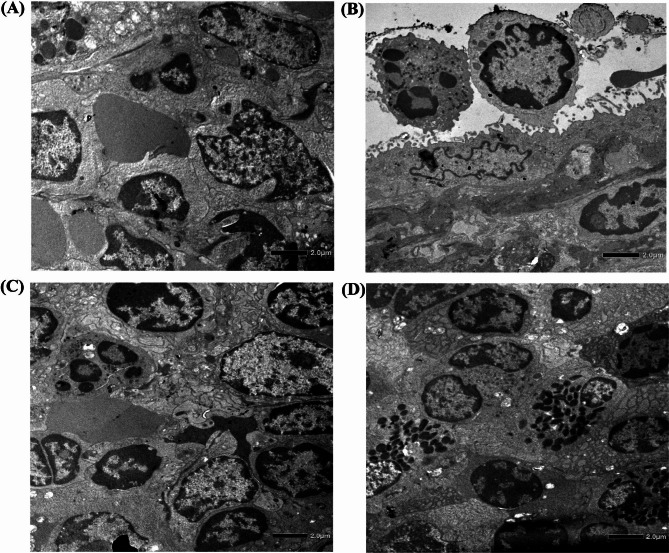
Transmission electron micrograph of mice spleen sections. Magnification, X 1500. **(A)** Control: without any treatment. Central venule, hepatic sinusoids, plates of hepatic cells, and portal areas were seen. **(B)** Infected: mice liver at 48 h post-challenge. The central venule was inflamed. **(C)** Prophylactic treatment grouped mice. **(D)** Therapeutic treatment grouped mice

The spleen is a large secondary lymphoid organ and it is composed of two compartments: red pulp and white pulp. Figure 6 showed the histopathological sections of the spleen tissue. A clear distinction between the red and white pulp, lymphoid follicles, and marginal zones was observed in the spleen of normal mice in Figs. 6A and 7A.

However, the *S.* Typhi*-*infected spleen in Figs. 6B and 7B showed severe congestion and enlarged red pulp, splenomegaly, inflamed central artery, lymphoid follicle hyperplasia, and eventually disruption of white pulp structure. But in the preventive group (Figs. 6C and 7C) and therapeutic group (Figs. 6D and 7D), significant changes were observed. In the therapeutic group, the white pulp structure was eventually disrupted but not as severe as in the infected group. In contrast, the preventive group showed less structural disorganization than the therapeutic group.

## Discussion

*S. enterica* contains over 2500 identified serotypes, and they differ significantly in terms of pathogenicity. Among them, *S.* Typhi is one of the major *Salmonella enterica* serovars that cause enteric fever or salmonellosis in humans. Additionally, it has the ability to produce biofilm, which is pathogenic in nature and also develops antibiotic-resistant bacteria. Antimicrobial medicines are mostly used to treat and prevent salmonellosis in humans and animals, they are nevertheless employed in certain nations to encourage the development of food animals [26]. In hospitals, communities, and livestock settings, their indiscriminate usage has facilitated the development of bacterial resistance. Either through the expansion of the resistant bacteria or through the transfer of resistance genes from animal to human, AMR can spread from animals to humans and vice versa [27]. Additionally, the development and spread of multidrug-resistant bacteria have provided a strong motivation to explore effective prophylactic and therapeutic means of eradication of bacterial infections. In this regard, the concept of phage therapy for controlling bacterial infections has been widely accepted.

With a population estimated to be > 10^30^, phages are the most prevalent biological species on earth [28]. Because of this, they can be investigated using several bacterial models and infect a wide range of bacterial species. For therapeutic purposes, tailed bacteriophages (dsDNA viruses) are recommended and they belong to a single order of Caudovirales. They typically contain a tail with or without tail fibers and a head or capsid with a dsDNA. During bacterial attachment, the phage uses its tail, tail fibers, or both to create precise interactions with the surface [29].

Studies from previous groups that used BALB/c mice as an infection model and oral administration of phages for therapy showed promising outcomes and phages were shown to cure bacterial infections more effectively [30]. Based on other reported studies, the phage STWB21 was also administered orally along with PBS buffer. A single dose of phage STWB21 at MOI 1 was given 60 min before and after infecting mice with *S.* Typhi bacteria resulting in the rescue of 66% and 33% of the mice, respectively. This was a remarkable result compared to the control group, which caused the death of all infected mice. The effect of phage STWB21 only and the PBS buffer showed no side effects on the health of the experimental mice groups. Thus, phage rescue experiments could be conducted without bias.

In this study, the most important finding was that the use of phage STWB21 has reduced the number of *Salmonella* Typhi colonies in mice in preventive as well as therapeutic treatment. These findings further suggest that phage STWB21 can both reduce the adversity of salmonellosis and control the transmission of the bacteria to the environment.

Previous studies from other groups reported that prophylactic bacteriophage administration is more effective than therapeutic administration in phage therapy using a single phage or phage cocktail [31, 32]. The current study also showed that the prophylactic administration of phage STWB21 decreases the colonization of a foodborne intestinal pathogen *S.* Typhi and represents a potential strategy to reduce the spreading of *Salmonella* Typhi during the outbreak.

In our previous study, we reported a T5-like phage STWB21, a member of the *Siphoviridae* family with a dsDNA of length 112,834 bp and a 40.37% GC content [25]. The phage STWB21 against pathogenic *S.* Typhi was found to successfully eliminate or reduce the bacteria in vitro. Here we are reporting the in vivo laboratory animal studies on the evaluation of the safety and effectiveness of this phage. BALB/c mice model system revealed promising results to be considered as important information for future clinical trials. Relentless efforts are being taken to use phage therapy during the post-antibiotic era and the current study is one such attempt for a better understanding of the usage of lytic phages as a therapeutic tool. However, the results of this study demonstrate that oral phage administration is significantly efficacious in the treatment of salmonellosis and other infections caused by *S.* Typhi.

## Materials and methods

### Bacterial strains and culture conditions

A clinically isolated *Salmonella* Typhi (Kol-551) strain was obtained from the Division of Bacteriology, ICMR-National Institute of Cholera and Enteric Diseases. The strain was preserved at − 80 °C in 8% glycerol-containing brain heart infusion broth (BHIB; BD Difco). The bacteria were grown on tryptic soy agar (TSA; BD Difco) and tryptic soy broth (TSB; BD Difco) at 37 °C.

### Phage preparation

*Salmonella* phage STWB21 isolated from the lake water of an outbreak area about 18 km from Kolkata, West Bengal, India. The phage sample was prepared by a double-layer agar method as previously described [25, 33]. Briefly, 250 µl of mid-log (exponential) phase liquid culture of *S.* Typhi was mixed with phage at a multiplicity of infection (MOI) of 0.01 and plated on soft agar (0.8%) overlay (3 ml) on nutrient agar and incubated at 37 °C. After that, the soft agar layer was scraped off when complete lysis occurred and suspended in 1 ml of Tris–MgCl_2_ buffer followed by centrifugation at 10,000 × g for 20 min at 4°C [34]. Then, the supernatant was purified and concentrated by ultracentrifugation (25,000 rpm, 1.5 h, 4 °C) and sucrose step-gradient ultracentrifugation (30,000 rpm, 2 h, 4 °C) respectively [35]. The concentrated phage STWB21 was preserved at 4 °C.

### Animal experiment

#### Selection of animals

BALB/c mice were collected from the NICED animal house facility to investigate the effect of phage STWB21 on *Salmonella* Typhi. Six weeks old, disease-free, healthy, active female and male BALB/c mice were chosen for the experimental purpose with animals weighing in the range of 22 ± 2 gm. Mice were fed sterile food and water *ad libitum*. Animals were kept for 10 days before experimentation to acclimatize to laboratory conditions. The animals were housed and the entire experiment was carried out in the animal house of ICMR-NICED.

#### Determination of LD_50_

To determine the LD_50_ of *Salmonella* Typhi (Kol-551), a total of thirty mice were distributed into five groups of six mice in each group and intraperitoneally injected with different *Salmonella* doses (from 1 × 10^4^ to 3 × 10^7^ colony-forming units/group). Six mice per group were kept in cages and challenged with above-mentioned inoculum of the *Salmonella* strain in respective groups. Mice were closely monitored and deaths of mice were noted during 7 days. The experiment was done in triplicates.

#### Efficacy of bacteriophage STWB21 in challenged BALB/c mice

To compare the efficiency of prophylactic versus therapeutic application of phage STWB21 on *S.* Typhi shedding, BALB/c mice were divided into five groups and each group consisted of 6 animals. The doses were fixed and prepared in PBS and administered. Mice from Group I received only PBS as a control, Group II animals were administered by intraperitoneal injection (i.p.) with *S.* Typhi (4.5 × 10^8^ CFU) diluted in PBS. Group III animals were orally administered with only STWB21 phage (1.5 × 10^10^ PFU) to check any lethal effect of phage on mice. Group IV mice represent the prevention group and Group V mice represent the treatment group. All five groups were kept in hygienic conditions with a continuous supply of food and water for 14 days. A significant observation was made and results were recorded (Table 1).

**Table 1:** Group-wise distribution of mice with intraperitoneal administration of bacteria and oral administration of phage

Group	Administration with various inducing agents
Group I (Receiving no treatment)	Mice + PBS
Group II (Infected with bacteria)	Mice + *S.* Typhi bacteria
Group III (Receiving only phage)	Mice + STWB21
Group IV (Prevention group)	Mice + STWB21 phage before 1 h infection + *S.* Typhi
Group V (Treatment group)	Mice + *S.* Typhi + STWB21 phage after 1 h infection

#### Enumerations of *Salmonella* and phages from mice tissues

Mice tissues (liver, and spleen) were collected on day two post-infection when the most prominent gut inflammation occurred [36]. Tissues were immediately weighed and homogenized using a bead-beating machine with 1.0 mm diameter silica beads (Biospec Products, Bartlesville, United States). *Salmonella* Typhi colony-forming unit (CFU)/gm tissues were determined by a serial dilution technique on the HEA agar.

#### H&E staining

At indicated time points, mice were euthanized and intestinal organs were carefully removed and fixed with 10% buffered formalin and embedded in paraffin. Six-micrometer sections were cut and stained with hematoxylin and eosin [37]. The stained sections were observed under a JENOPTIK GRYPHAX digital microscope.

#### Preparation and imaging of tissue sections by electron microscopy

For transmission electron microscopy (TEM) analysis, liver and spleen tissues were cut into 1 mm x 1 mm pieces and further processed following the previously described method [38]. Tissues were fixed in cacodylate-buffered glutaraldehyde at 4 °C overnight. After that, tissues were then post-fixed in 1% osmium tetroxide followed by dehydration with a graded series of ethanol (25%, 50%, 75%, and 100%). Then the samples were embedded in resin Agar100 and polymerization was done overnight at 60 °C. Gold sections of 60–90 nm were cut using a Leica Ultracut UCT ultramicrotome (Leica Microsystems, Germany) fitted with a Diatome diamond knife (Diatome, Hatfield, PA). Then the sections were transferred to 200 mesh copper or nickel grids (EMS). Next, the sections were dual-stained with 2% aqueous uranyl acetate and 0.2% lead citrate, and grids were air-dried. Grids were imaged using a JEOL 1400 electron microscope at an accelerating voltage of 120 kV.

### Statistical analyses

Data were expressed as mean ± standard deviation (SD). Three technical and three biological repeats were carried out, as needed. GraphPad Prism version 5 was used to carry out all the statistical analyses. Statistical significance was assessed using two-way ANOVA (*P < 0.05; **P < 0.01).

## Conclusion

In our previously published research, we found that the phage STWB21 against pathogenic *S.* Typhi successfully eliminated or reduced the bacterial population in vitro. These findings led us to undertake studies on the impact of the phage STWB21 in vivo. The results of the current investigation showed that phage STWB21 can prevent and treat *Salmonella* invasion in mouse models. The clinical significance of this phage to control *S*. Typhi-induced illnesses is further supported by the possibility of the oral administration of phage treatment. It may be advantageous in both preventive and therapeutic treatment of salmonellosis caused by *S.* Typhi without developing any adverse effects.

## Data Availability

The dataset used and/or analyzed during the current study is added in the manuscript. Additional datasets are not available from the authors.

## Abbreviations

LB: Luria Bertani broth
MOI: Multiplicity of infection
HEA: Hektoen enteric agar
TEM: Transmission electron microscopy
CFU: Colony-forming units
PFU: Plaque-forming units
PBS: Phosphate-buffered saline
